# Immunological Markers for Central Nervous System Glia

**DOI:** 10.1007/s12264-022-00938-2

**Published:** 2022-08-26

**Authors:** Hao Huang, Wanjun He, Tao Tang, Mengsheng Qiu

**Affiliations:** 1 Zhejiang Key Laboratory of Organ Development and Regeneration, Institute of Life Sciences, College of Life and Environmental Sciences, Hangzhou Normal University, Hangzhou, 311121 China; 2 Department of Anatomy, Cell Biology and Physiology Stark Neurosciences Research Institute, Indiana University School of Medicine, Indianapolis, IN 46202 USA

**Keywords:** Glial cells, Oligodendrocytes, Astrocytes, Microglia, Markers

## Abstract

Glial cells in the central nervous system (CNS) are composed of oligodendrocytes, astrocytes and microglia. They contribute more than half of the total cells of the CNS, and are essential for neural development and functioning. Studies on the fate specification, differentiation, and functional diversification of glial cells mainly rely on the proper use of cell- or stage-specific molecular markers. However, as cellular markers often exhibit different specificity and sensitivity, careful consideration must be given prior to their application to avoid possible confusion. Here, we provide an updated overview of a list of well-established immunological markers for the labeling of central glia, and discuss the cell-type specificity and stage dependency of their expression.

## Introduction

Glial cells, or glia, were first described by neuroscientists including Rudolf Virchow, Santiago Ramón y Cajal, and Pío del Río-Hortega over a century ago [1]. With time, it was demonstrated that glial cells are diverse in type and function. It is now clear that these cells constitute more than half of the total number in the mammalian central nervous system (CNS) [2, 3]. Central glia can be classified as macroglia, which refers to both astrocytes and oligodendrocytes, and microglia. They have distinct embryonic origins, developmental trajectories, and functions. During early CNS development, neural progenitor cells or radial glial cells (RGCs) first give rise to neurons, followed by glial progenitor cells. Glial progenitors then produce either oligodendrocyte progenitor cells (OPCs) or astrocyte precursor cells (APCs), which subsequently undergo a series of morphological and molecular changes to become functionally mature oligodendrocytes or astrocytes [4]. In contrast, microglia are CNS-resident macrophages which originate from the yolk sac and fetal liver during early embryogenesis [5, 6].

In the vertebrate CNS, oligodendrocytes form myelin sheathes around axons to facilitate the rapid propagation of action potentials and provide trophic support for the myelinated axons [7]. Deficits of myelin are found in many neurological diseases, such as multiple sclerosis and the leukodystrophies [8]. The development of oligodendrocytes is coordinated by a large cohort of intracellular factors and extracellular signals [9, 10]. In mouse spinal cord, OPCs are initially generated from the OLIG2+ ventral neuroepithelial cells of the motor neuron progenitor (pMN) domain around embryonic day 12.5 (E12.5), and later from the ASCL1+ dp3-dp5 neuroepithelial domains in the dorsal spinal cord at ~ E14.5 [11, 12]. In the embryonic telencephalon, the first group of OPCs originate from the NKX2.1+ medial ganglionic eminence (MGE) and anterior entopeduncular area (AEP) in the ventral forebrain at E12.5–E14.5. The second wave of OPCs are generated from the lateral (LGE) and/or caudal ganglionic eminences at E14.5–E16.5. Starting at ~ E17.5, neural progenitors in the dorsal cortical region give rise to the third wave of OPCs [13]. The generation of OPCs follows a chronological order from ventral to dorsal along the entire anterior-posterior axis. Once generated, OPCs proliferate rapidly and migrate into the surrounding regions. OPCs start to differentiate into newly-formed oligodendrocytes (NFOs) at E18 in the spinal cord and at ~ P2–P3 in the forebrain. NFOs begin to contact and wrap around neuronal axons, express myelin proteins; and further differentiate into mature oligodendrocytes elaborating compact myelin sheaths. Notably, OPCs display significant functional redundancy throughout the CNS both during development and in adulthood, as the OPCs eliminated in one region are quickly replenished by OPCs from surrounding areas [13–15]. Studies over the past several decades have successfully identified a large number of stage-specific oligodendrocyte markers, critical tools for investigating the molecular and genetic control of oligodendrocyte development and axonal myelination in health and disease [16].

Astrocytes are the most functionally diverse glial cells and are involved in nearly all aspects of CNS physiology and functioning. The functional roles of astrocytes include but are not limited to maintaining the blood-brain barrier (BBB), providing physical and trophic support to neurons, axon guidance, synapse formation and remodeling, modulating synaptic transmission, regulating osmotic pressure, and ion homeostasis [17]. As noted earlier, APCs also originate from late RGCs in the ventricular zone of the CNS after neurogenesis. At the early stage of neural development, APCs undergo rapid local proliferation to expand their population. However, there are several unique features in the development of astrocytes as compared to that of oligodendrocytes. First, astrocytes arise from nearly all of the ventricular regions in the CNS and migrate to their final destinations in the direction of their radial glial processes [17]. Second, there is no functional redundancy among astrocytes in different regions [18]. Astrocytes are allocated to spatial regions in accordance with their sites of embryonic origin in the ventricular zone (VZ), and do not migrate tangentially to adjacent areas throughout life, even after acute CNS injury [19]. Third, RGCs, APCs, and astrocytes share many of the same molecular markers, such as GFAP and ALDH1L1 [20, 21]. Therefore, unlike the development of oligodendrocytes, there are few definite stage-specific molecular markers for the astrocyte lineage, and transcription factors that control the differentiation and maturation of astrocytes have yet to be found. Based on these and other findings, we propose that astrocytes are dormant neural progenitor cells that are influenced by local environments and are functionally adapted to support the local neuronal populations [22].

Unlike oligodendrocytes and astrocytes that are derived from neural progenitor cells, microglia are CNS-resident macrophages of blood origin. The CNS macrophages comprise microglia and border-associated macrophages (BAMs, also termed CNS-associated macrophages) in the meninges, choroid plexus, and perivascular spaces. Although microglia have been studied for decades, their developmental origin has been under debate for quite a long time. Now it is known that the bulk of microglia originate from hematopoietic progenitors in the yolk sac and enter the CNS tissue during early embryonic development [23, 24]. With the closure of BBB, renewal of the microglial population in the CNS mainly depends on their own proliferation [25]. With the aid of a number of cell-specific markers, microglia have been found to play critical roles in the development and functional maintenance of the CNS, including synaptic elimination, neural circuit wiring, axon tract fasciculation, vasculature tip cell fusion, synapse pruning, and inflammation [26].

In the past decade, a significant progress has been made in the studies of glial development, homeostasis, reactivation, regeneration, and other functions in the CNS. Clearly, these studies are highly dependent on the proper use of specific molecular markers for particular developmental stages or functional states. Theoretically, the most important features for a “perfect” marker gene are its high specificity and affinity. However, marker genes often display varying degrees of specificity and efficiency, and they even vary at different developmental stages or between species. Therefore, the proper use of cellular markers is critical for drawing correct conclusions.

Here, we provide a comprehensive and updated review of a list of well-characterized glial markers and discuss the pros and cons of these markers in their applications (Table 1).

**Table 1:** Cellular markers for glial cells

Markers	Applications	Specificity and efficiency
SOX10	Pan-oligodendrocyte lineage cells	Excellent
OLIG2	Pan-oligodendrocyte lineage cells	Also expressed in NPCs in the ventral forebrain and pMN domain of spinal cord at embryonic stage, cortical MIPCs and brain astrocyte before weaning stage
PDGFRA	OPCs	Excellent in rodents, weakly expressed in human cortical MIPCs
NG2	OPCs	Also labels vascular pericytes, especially in embryonic and newborn CNS
CC1/QKI7	NFOs and mature OLs	Expressed in NPCs and immature macroglial cells in neonatal CNS
MYRF	NFOs and mature OLs	Excellent
ENPP6, BMP4	NFOs	Perfect, but lack of antibodies
O4	Pre-myelinating OLs	Perfect, but is not suitable for counting *in vivo*
NKX2.2	Pre-myelinating OLs	Fine for *in vivo* labeling, also expressed in some neurons
CNP, MBP, PLP, MAG, MOG	NFOs and mature OLs	Excellent. Reflects the biomass of myelin sheaths by immunostaining, and ISH is required for cell counting (except MBP)
CAII	Type I/II OLs	Specific to mature OLs myelinating small diameter axons
GFAP	White matter astrocytes and reactive astrocytes	Also expressed in late RGCs
S100B	Gray matter astrocytes	Does not label white matter astrocytes, also expressed in differentiating oligodendrocytes. Not expressed in RGCs and newborn astrocytes
ALDH1L1, ACSBG1	Pan-astrocyte lineage	Also expressed in RGCs
SOX9, NFIA	Pan-astrocyte lineage	Also expressed in RGCs and OPCs; suitable for cell counting
FABP7	Pan-astrocyte lineage	Also expressed in RGCs; transiently and weakly expressed in OPCs
GLAST	Pan-astrocyte lineage	Transmembrane protein; also expressed in RGCs
GS	Pan-astrocyte lineage	Also expressed in RGCs and myelinated OLs
AQP4	Astrocyte end-feet	Not expressed in RGCs and newborn astrocytes. AQP4 is concentrated in astrocytic end-foot membranes surrounding blood vessels
IBA1, CX3CR1	Pan-microglia	Also expressed in macrophages
P2RY12, TMEM119	Resting microglia	Not expressed in macrophages
CD68	Reactive microglia	Dotted distribution of immunostaining signal
CD206, LYVE1	Macrophages including BAMs	Not expressed in microglia
HEXB, Siglec-H	Pan-microglia	Not expressed in macrophages including BAMs

NPCs, neural progenitor cells; OPCs, oligodendrocyte progenitor cells; NFOs, newly-formed oligodendrocytes; pMN, motor neuron progenitor domain; MIPCs, multipotent intermediate progenitor cells; CNS, central nervous system; OLs, oligodendrocytes; RGCs, radial glial cells; BAMs, border-associated macrophages

## Markers for Cells of Oligodendrocyte Lineage

### SOX10

**Fig. 1:**
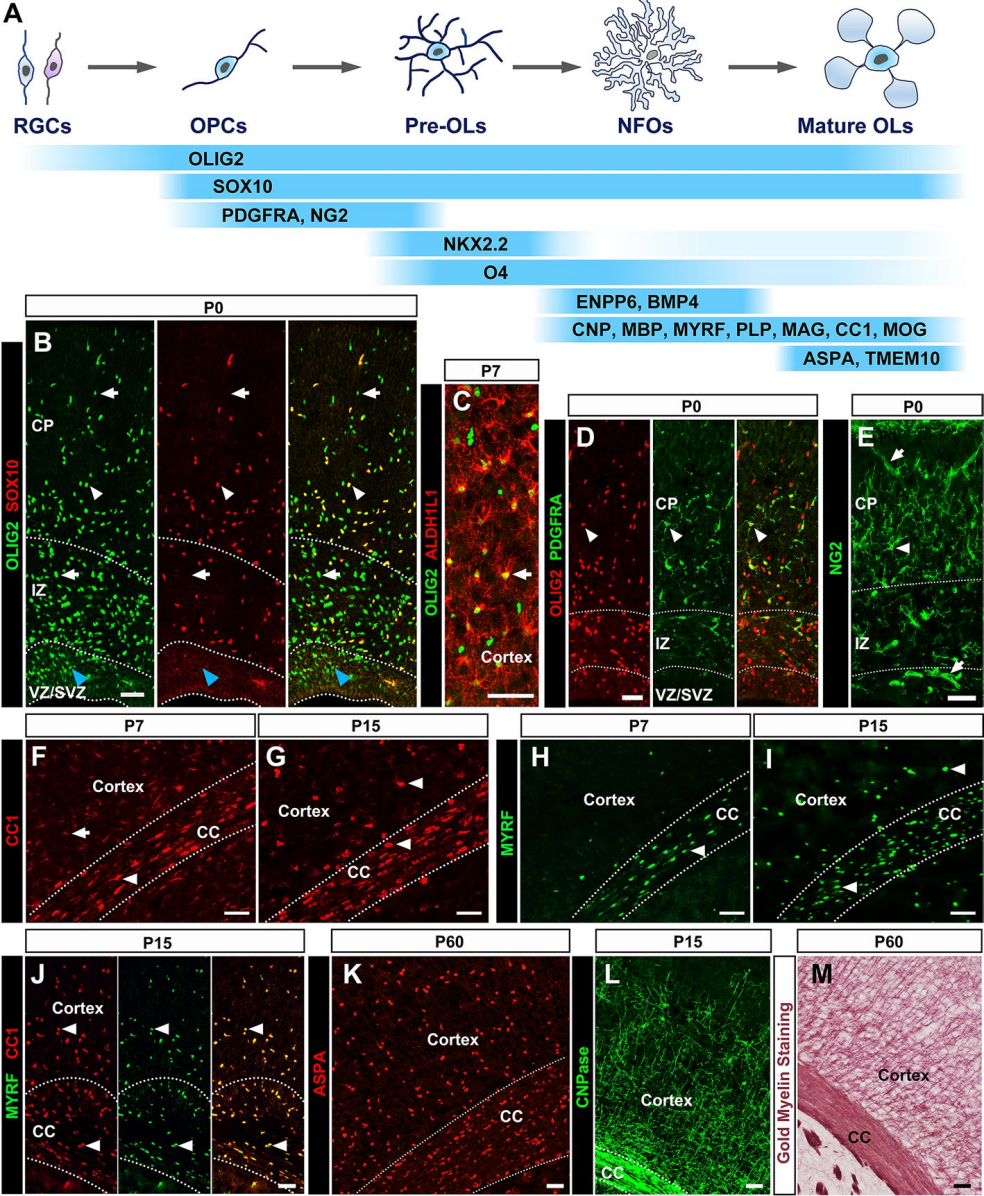
Molecular markers for cells of oligodendrocyte lineage. **A** Diagram of oligodendrocyte development and stage-specific markers. **B** OLIG2+ cells include SOX10+ OPCs (white arrowheads), SOX10- astrocytes (arrows), and MIPCs in the SVZ of P0 mouse cortex (blue arrow heads). **C** Nearly all ALDH1L1+ astrocytes express OLIG2 in P7 mouse cortex (arrows). **D**, **E** PDGFRA and NG2 label OPCs in P0 cortex (arrowheads). Note that NG2 is also expressed in vascular pericytes (arrows in **E**), while PDGFRA is not. **F**–**J** CC1 but not MYRF is weakly expressed in astrocytes in P7 cortex (arrows). At P15, all of the CC1+ cells are MYRF+ differentiated oligodendrocytes (arrowheads). **K**, **L** Expression patterns of ASPA and CNPase in mouse cortex. **M** Brain myelin structures visualized by gold-based staining. NPCs, neural progenitor cells; OPCs, oligodendrocyte progenitor cells; Pre-OLs, pre-oligodendrocytes; NFOs, newly-formed oligodendrocytes; OLs, oligodendrocytes; VZ/SVZ, (sub)ventricular zone; IZ, intermediate zone; CP, cortical plate; CC, corpus callosum. Scale bars, 50 μm.

The SRY-box transcription factor SOX10 is one of the earliest identified pan-oligodendroglial markers and is exclusively expressed in cells of the oligodendrocyte lineage in the CNS [27]. Genetic studies revealed that SOX10 plays a central role in controlling oligodendrocyte development, as conventional deletion of the *Sox10* gene leads to a dramatic delay of OPC differentiation with little impact on OPC generation and distribution in spinal tissue [28]. However, *Sox10*-KO enhances the phenotype of *Sox9* mutants in OPC generation [29, 30], while the double knock-out of *Sox8* and *Sox10* genes in differentiated oligodendrocytes results in demyelination in the mouse CNS [31, 32]. These findings suggest that SOX10 regulates nearly all stages of oligodendrocyte development, including their initial generation from neural progenitors, terminal differentiation, and myelin maintenance. As a specific marker for the oligodendroglial lineage, SOX10 is activated in OPCs immediately after their fate specification in the ventricular regions (Fig. 1A, B). SOX10 expression is maintained in OPCs and markedly upregulated at the onset of OPC differentiation. It is worth highlighting that Sox10 is considered to be the most reliable pan-oligodendrocyte marker in both brain and spinal cord, due to its specificity and high efficiency in labeling all cells of oligodendrocyte lineage throughout the life span.

### OLIG2

This gene is probably the most frequently-used marker for cells of oligodendrocyte lineage, partly due to its commercial availability. However, OLIG2 is not always a reliable marker for labeling oligodendroglia. In the spinal cord, OLIG2 expression is initially activated in neural progenitors of the pMN domain at ~ E8.5, but is rapidly lost from their motor neuron progeny during neurogenesis [33, 34]. As development proceeds, OLIG2 expression is retained in OPCs but is slightly downregulated in differentiated oligodendrocytes [35, 36]. In the brain, OLIG2 is broadly expressed in the VZ of the ventral telencephalon, including the LGE, MGE, and AEP regions at embryonic stages [37–39]. In the dorsal cortical region, the earliest OLIG2-positive cells appear at ~ E17.5, coincident with the onset of local gliogenesis [40, 41]. These OLIG2+ pluripotent precursor cells, termed multipotent intermediate progenitor cells (MIPCs) (also named pri-OPCs or pre-OPCs in other studies [42, 43]), are the common precursors of cortex-derived astrocytes, oligodendrocytes, and olfactory bulb interneurons (OBiNs) [40, 44] (OLIG2+ cells in the VZ and subventricular zone (SVZ), Fig. 1B). Despite its rapid downregulation in OBiNs, OLIG2 expression is sustained in glial cell progeny, including newborn astrocytes (APCs) and OPCs [40, 44, 45] (Fig. 1B, C). In the cortical region of neonatal mice, nearly all astrocytes are immunoreactive to OLIG2 until P7 (Fig. 1C). Within the next week or so, the expression level of OLIG2 is rapidly downregulated as they progress further along the astrocyte lineage. By the age of animal weaning, ~ 99% of the OLIG2+ cells in cortical tissue co-express SOX10, and *vice versa*, indicating that OLIG2 predominantly labels oligodendrocyte cells at this stage [46] (Fig. 1B). In summary, OLIG2 is an excellent pan-oligodendroglial marker in the spinal cord, since only a small fraction of pMN-derived astrocytes expresses this gene during embryonic stages [47, 48]. However, OLIG2 cannot be used as a specific oligodendrocyte marker in embryonic or early postnatal brain tissue. Consistent with the regional difference in OLIG2 expression, deletion of the *Olig2* gene affects the development of cortical astrocytes [45, 49], but not that of spinal astrocytes [50, 51].

### PDGFRA and NG2

Platelet-derived growth factor α receptor (PDGFRA) and NG2 proteoglycan (also known as chondroitin sulfate proteoglycan 4) are commonly-used markers for OPCs in the CNS. In rodents, PDGFRA is exclusively expressed in OPCs from their origin from neural progenitor cells in both the embryonic and adult CNS (Fig. 1D) [52, 53]. The function of the PDGFA/PDGFRA signaling pathway is to stimulate OPC proliferation, but meanwhile to inhibit their differentiation. Ablation of either *Pdgfa* or *Pdgfra* at embryonic stages causes premature differentiation of OPCs, resulting in severe hypomyelination due to the reduced proliferation and faster depletion of the progenitor pool [54, 55].

NG2 is another widely-used OPC marker, and NG2+ glial cells produce myelinating oligodendrocytes in adult CNS tissue [46, 56–58]. However, unlike PDGFRA, NG2 is also expressed in vascular pericytes, especially in the embryonic and newborn brain (Fig. 1E) [59, 60]. In general, PDGFRA is a more reliable OPC marker than NG2 for both *in situ* hybridization and immunostaining, and the pericytes must be culled when NG2 is used in labeling OPCs.

### NKX2.2

Homeodomain transcription factor NKX2.2 is selectively upregulated in differentiating OPCs or pre-oligodendrocytes, but is rapidly down-regulated after oligodendrocyte differentiation [61]. Genetic studies have demonstrated that NKX2.2 controls the timing of OL differentiation, as conditional deletion of *Nkx2.2* causes a developmental delay of OL differentiation [62], and overexpression of NKX2.2 in OPCs results in precocious OL differentiation [55]. NKX2.2 promotes oligodendrocyte differentiation by directly inhibiting *Pdgfra* expression. However, NKX2.2 is also expressed in a subset of neurons in the ventral spinal cord and ventral thalamus [63–65]. Thus, identification of pre-myelinating oligodendrocytes by NKX2.2 expression in the gray matter must be combined with other lineage-specific transcription factors such as OLIG2 or SOX10.

### CC1

The monoclonal antibody anti-adenomatous polyposis coli (APC) clone CC1 is widely used to mark differentiated/mature oligodendrocytes without labeling myelin. However, previous studies have shown that the CC1 antibody does not bind APC [66]. In fact, the expression of APC is completely different from that of CC1 [66–69]. It is now clear that the CC1 antibody recognizes a specific isoform of the RNA-binding protein Quaking (QKI, encoded by the *Qk* gene), namely QKI7 [69]. During early development, *Qk* is broadly expressed in neural progenitor cells and later in their glial progeny, including OPCs and newborn APCs (Fig. 1F) [70]. With time, *Qk* expression is gradually reduced in astrocyte lineages, but is strongly upregulated in differentiated oligodendrocytes (Fig. 1G, J) [71]. As an antibody recognizing the cytoplasmic isoform QKI7 of QK proteins, CC1 is a useful and reliable marker for counting the number of mature oligodendrocytes in postnatal animals (older than P15 in the brain and P7 in the spinal cord). When used in the CNS of neonatal mice, astrocytes and OPCs with weak CC1 expression should be excluded from the mature oligodendrocyte population based on their morphology, gene expression level, and tissue distribution.

### Myelin Proteins

Myelin is made of lipids (>70% of its dry weight) and proteins. Myelin proteins play important roles in cell adhesion, axon-myelin interactions, and the integrity of compact myelin structure. The most abundant proteins in CNS myelin sheaths include PLP (proteolipid protein 1), MBP (myelin basic protein), CNP (2′,3′-cyclic nucleotide 3′-phosphodiesterase, CNPase), MAG (myelin-associated glycoprotein), and MOG (myelin oligodendrocyte glycoprotein) [72]. These proteins constitute >30% of the total myelin-associated proteins [8]. Detection of their expression at the RNA or protein level directly monitors the differentiation state of oligodendrocytes both *in vitro* and *in vivo*. These myelin genes are activated when OPCs start to differentiate, and are sustained in myelinating and myelinated cells. The expression of myelin genes commences at ~E18 in mouse spinal cord or P2–P3 in the cortex by RNA *in situ* hybridization, but their proteins can only be detected ~ 2 days later. Generally speaking, the chronological order of expression of these markers *in vivo* is CNP/MBP, PLP/MAG, MOG/CC1, although the temporal difference is quite small. It should be noted that myelin proteins are predominantly localized in the myelin processes of oligodendrocytes. Thus, immunostaining against these proteins in CNS tissue usually reflects the biomass of myelin sheaths, and is not suitable for cell counting (Fig. 1L). Should the quantitative analysis of cell numbers be required, RNA *in situ* hybridization of these myelin genes (except for *Mbp* mRNA, which is distributed in cellular processes as well) would be a better choice.

### MYRF

The myelin regulatory factor gene (MYRF) is a novel type of membrane-bound transcriptional factor that is evolutionarily conserved from invertebrates to vertebrates. It is initially synthesized as a type-II membrane protein, which subsequently undergoes homo-trimerization and self-cleavage on the endoplasmic reticulum (ER) membrane. Once cleaved on the ER membrane, the N-terminal trimers of MYRF are released and translocate into the nucleus to function as a transcriptional activator [73, 74]. As the downstream target of SOX10, MYRF is a key regulator of myelin gene expression, which is essential for myelin formation and maintenance [75]. In the CNS, MYRF expression is strictly restricted to oligodendrocytes. Its expression is selectively upregulated in oligodendrocytes at the beginning of cell differentiation, and sustained in mature oligodendrocytes, in a pattern similar to that of myelin genes such as *Plp* and *Mbp* (Fig. 1H–J) [75, 76]. Conditional ablation of *Myrf* in OPCs blocks myelinogenesis during development or myelin maintenance/repair in adulthood [76]. Given its higher specificity and nuclear localization, MYRF is considered to be a better marker than CC1 for differentiated oligodendrocytes and cell counting.

### Other Oligodendroglial Markers

In addition to the markers described above, a number of others are also frequently used to identify cells of oligodendrocyte lineage in a stage-specific manner. For instance, the monoclonal antibody O4, like NKX2.2, preferentially labels immature differentiating oligodendrocytes (or pre-oligodendrocytes) before the expression of MBP and PLP [64, 77, 78]. This mouse IgM antibody works well in both culture and tissue sections. Besides, *Enpp6* and *Bmp4* have recently been shown to selectively label newly-formed oligodendrocytes, but their expression is downregulated in more mature myelinating oligodendrocytes [79, 80]. Unfortunately, working antibodies have not been developed for these proteins. As oligodendrocytes undergo terminal maturation, they start to express Opalin (also known as TMEM10) and ASPA (aspartoacylase) [81–84]. The expression of these two new markers in mature oligodendrocytes occurs several days later than that of MBP and PLP *in vivo*, *i.e.* at ~ P5–P7 in spinal tissue or P10–P14 in the corpus callosum. For identification of more mature oligodendrocytes, ASPA seems to be the best choice due to its preponderant localization in the cell body (Fig. 1K). Another marker for mature oligodendrocytes is CAII (carbonic anhydrase 2), and this gene is a specific marker for type I/II oligodendrocytes which are predominantly myelinate small-diameter axons [85, 86]. In addition, a simple myelin staining method, Black-Gold, was developed some years ago, based on the specific affinity of gold phosphate complex with lipidic myelin structures [87]. This chemical staining method is relatively fast and simple compared to immunostaining, and produces much higher resolution than the traditional Luxol Fast Blue staining (Fig. 1M).

## Markers for Cells of Astrocyte Lineage

### GFAP

Glial fibrillary acidic protein (GFAP) was one of the first identified astrocyte markers [88, 89]. For decades, GFAP has been widely used as the standard astrocyte marker in numerous studies because of its robust staining and the lack of better markers. GFAP labels cultured astrocytes *in vitro* and reactive astrocytes in injured or pathological CNS tissues. In normal CNS tissue, GFAP is predominantly expressed by astrocytes in the white matter (fibrous astrocytes) and the parenchyma region near the leptomeninges, but very weakly in gray matter protoplasmic astrocytes. However, GFAP also labels the late radial glial cells in embryonic cortical tissue [40]. It is worth mentioning that GFAP has a high degree of homology to other types of neurofilament proteins (*e.g.* Nestin and Vimentin). Therefore, the specificity of GFAP antibodies must be carefully characterized, due to the potential cross-reactivity issue.

### S100B

**Fig. 2:**
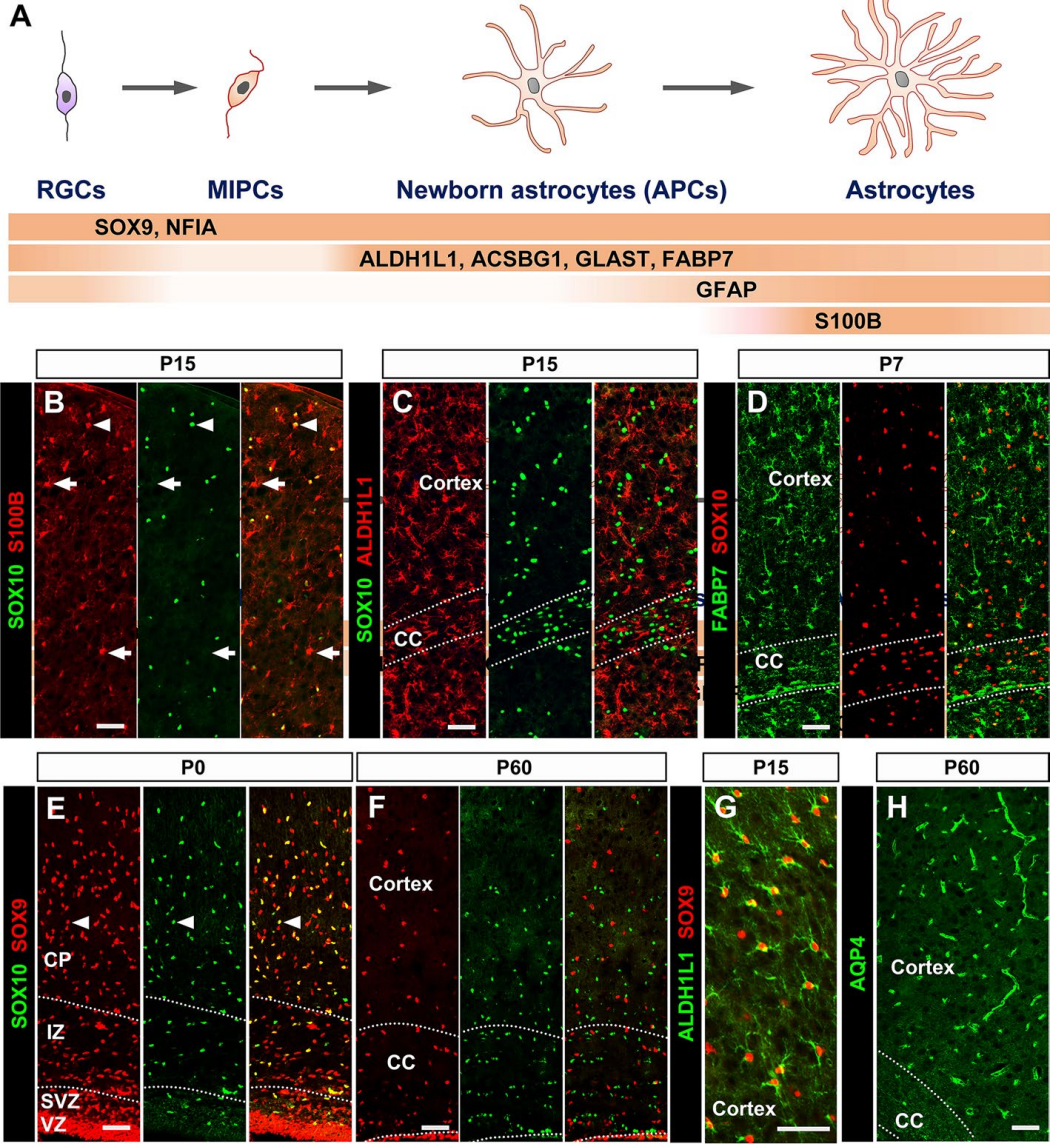
Cellular markers for cells of astrocyte lineage. **A** Diagram of astrocyte development and the expression of specific markers. **B** Besides gray matter astrocytes (arrows), many S100B+ cells are co-labeled with the pan-oligodendrocyte marker SOX10 (arrowheads). **C** ALDH1L1 expression is only detected in astrocytes, but not in SOX10+ oligodendrocytes. **D** FABP7 is an operational astrocyte marker. **E**, **F** Nearly all of SOX10+ OPCs co-express SOX9 in P0 forebrain (arrowheads), and SOX9 primarily marks astrocytes in the adult brain. **G** All ALDH1L1+ astrocytes co-express SOX9 in the brain. **H** AQP4 is localized in astrocyte process, and APQ4 immunolabeling reveals the entire network of vessels covered by astrocytic end-feet. RGCs, radial glial cells; APCs, astrocyte progenitor cells; VZ/SVZ, (sub)ventricular zone; IZ, intermediate zone; CP, cortical plate; CC, corpus callosum. Scale bars, 50 μm.

Besides GFAP, S100B (S100 protein, beta polypeptide, neural; S100β) has also been widely used for decades as an astrocyte biomarker in both the developing and adult CNS [90, 91]. S100B is a Ca^2+^-binding protein, and has been implicated in several neurological diseases including Alzheimer’s disease, Parkinson’s disease, and neuropathic pain [92]. However, emerging evidence suggests that S100B is also expressed in cells of oligodendrocyte lineage [93, 94]. Our recent study demonstrated that the earliest S100B+ cells are differentiating oligodendrocytes before P4 in the forebrain, rather than astrocytes as previously thought. Its expression in cortical gray matter astrocytes occurs only after P4, a time-point when astrocytes have already migrated from the germline region [95]. Unexpectedly, we found that almost all of the S100B+ cells in the white matter are SOX10+/MYRF+ oligodendrocytes from the neonatal stage to adulthood. In the postnatal CNS, S100B marks protoplasmic astrocytes in the gray matter and maturing oligodendrocytes in both the gray and white matter (Fig. 2B). Nevertheless, S100B still holds advantages as a marker for astrocytes. First, nearly all protoplasmic astrocytes in the gray matter express S100B in post-weaning animals. Second, S100B protein is predominantly localized in the cell body and thus highly suitable for cell counting. Third, unlike other astrocyte markers, S100B is not expressed by radial glial progenitor cells and newborn astrocyte progenitors (Fig. 2A) [95]. Thus, S100B remains an excellent choice for gray matter astrocytes when combined with SOX10 to exclude its expression in oligodendrocytes.

### ALDH1L1 and ACSBG1

Recent studies on astroglial gene expression profiling have identified two new astroglial marker genes, *Aldh1l1* and *Acsbg1*. *Aldh1l1* (aldehyde dehydrogenase 1 family, member L1) encodes a folate enzyme of tetrahydrofolate synthesis. Several studies have demonstrated the specific expression of ALDH1L1 in astrocytes [96, 97]. We have also confirmed that ALDH1L1+ cells out of the germline region never co-express the pan-oligodendroglial marker SOX10 in both the embryonic and adult brain, indicating their astrocyte identity (Fig. 2C) [95]. ACSBG1, a protein with very long-chain acyl-CoA synthetase activity, has been reported to display an expression pattern similar to that of ALDH1L1 [97]. However, detailed analysis of its spatiotemporal expression pattern is still required to validate its specificity in the astrocyte lineage. In addition, it has been noted that the expression of ALDH1L1 decreases in adulthood [96], calling for better astrocyte markers for the adult CNS.

### SOX9 and NFIA

To date, the transcriptional regulation of astrocyte development is still largely unknown. The SRY-box transcriptional factor SOX9 was originally reported to determine glial fate choice in the developing spinal cord. SOX9 is initially expressed in neural progenitor cells, and later in glial cells in the marginal zone, including astrocytes and OPCs (Fig. 2E) [29]. Intriguingly, SOX9 expression is gradually downregulated in the oligodendrocyte lineage as development proceeds and becomes progressively restricted to astrocytes in the adult mouse CNS (Fig. 2F, G) [29, 98]. NFIA (nuclear factor I/A) is a target transcriptional factor of SOX9 and has been implicated in the regulation of astrogliogenesis [99, 100]. Although the expression pattern of NFIA in glial cells is quite similar to that of SOX9, it is also expressed by motor neurons in the spinal cord and deeper layer projection neurons in the cortex [101]. As NFIA down-regulation in oligodendrocytes occurs much slower than SOX9, a higher proportion of SOX10+ OPCs is still more immunoreactive to NFIA than to SOX9 in the adult mouse CNS. Therefore, SOX9 and NFIA could serve as pan-astrocyte markers due to their high immunoreactivity and nuclear localization, but only when OPCs are excluded.

### Other Astrocyte Markers

A number of other astrocytic markers have also been described. GLAST and GLT-1, also known as EAAT1/SLC1A3 and EAAT2/SLC1A2, respectively, are the primary astrocytic glutamate transporters in the adult CNS, accounting for > 90% of synaptic glutamate clearance [102, 103]. While the GLAST expression pattern is consistent with a pan-astrocyte marker, GLT-1 is also expressed in neurons in both the brain and the spinal cord [104, 105]. As a transmembrane protein, GLAST immunostaining displays punctate/reticular-like structures and is not suitable for outlining the shape of astrocytes *in vivo*, especially in adult animals. Nonetheless, we found that GLAST specifically labels cultured astrocyte *in vitro*. Glutamine synthase (GS, Glul) is another commonly-used astrocyte marker [106]. However, it has recently been reported that GS is activated in mature oligodendrocytes in mouse brain and spinal cord [107, 108]. The onset of GS expression in mature oligodendrocytes is between P21 and P28 in mouse brain, which is later than the appearance of the mature oligodendrocyte marker ASPA. FABP7 (fatty acid binding protein 7, also known as BLBP) has occasionally been used to label astrocytes in some studies [109]. It has also been suggested that FABP7 is expressed in OPCs in both mouse and chicken CNS during development and in adulthood [110]. Nevertheless, FABP7 can still be used as an approximate astrocyte marker, since its expression in OPCs is very weak (Fig. 2D). Other astrocytic markers include Aldolase C (ALDOC), AQP4 (aquaporin 4) (Fig. 2H), and the newly-described transcriptional factor ZBTB20 [111–113]. However, more detailed analyses with molecular and genetic approaches are needed to determine the specificity and expression dynamics of these markers.

### Markers for Microglia

**Fig. 3:**
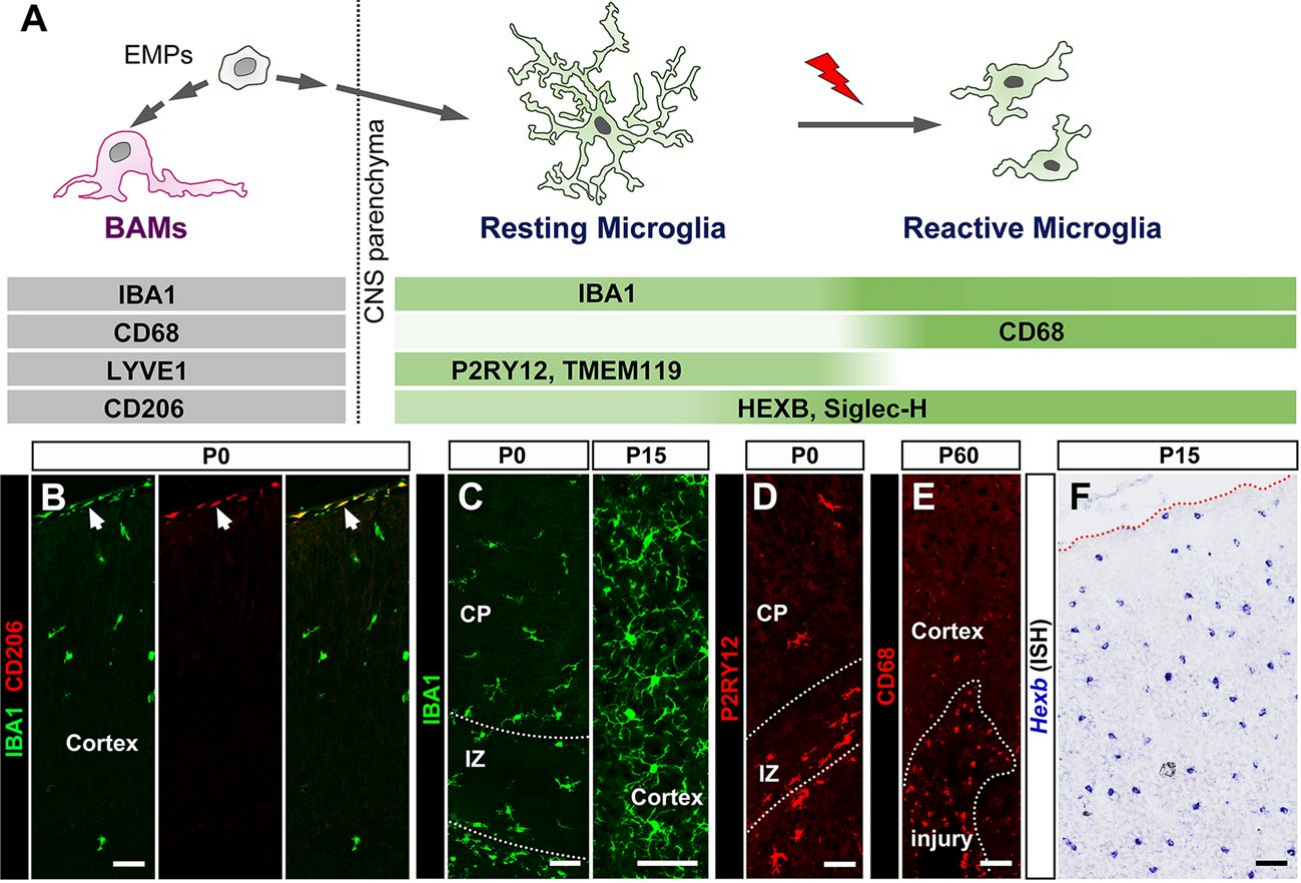
Molecular markers for microglia. **A** Diagram of microglial development and lineage-specific markers. **B** CD206 marks BAMs (arrows) but not microglia. **C** IBA1 expression in microglia in P0 and P15 mouse brain. **D** P2RY12 expression in microglia of P0 cortex. **E** CD68 labels reactive microglia after CNS injury. **F** Expression of *Hexb* mRNA in microglia revealed by *in situ* hybridization. BAMs, border-associated macrophages; VZ/SVZ, (sub)ventricular zone; IZ, intermediate zone; CP, cortical plate. Scale bars, 50 μm.

CNS macrophages consist of two distinct types of cell, microglia in the parenchyma and border-associated macrophages (BAMs) in the meninges, choroid plexus, and perivascular spaces. Both cell types are derived from erythromyeloid progenitors during early embryonic development (Fig. 3A) [23, 114]. Therefore, it is not surprising that microglia share many of the same molecular markers with BAMs. It is well documented that two functional states are associated with microglia: resting microglia with ramified morphology, and reactive microglia with an amoeboid shape in response to a pathological environment. In the previous studies, several molecular markers have been identified that can discern microglia from BAMs, or resting from reactive microglia.

### IBA1 and CX3CR1

To date, the most reliable and effective method for detection of microglia relies on the discovery of IBA1 protein by a Japanese group in 1996 and the demonstration of its microglial specificity [115, 116]. IBA1 protein, encoded by the *Aif1* gene (allograft inflammatory factor 1), was initially reported to be involved in the membrane ruffling and phagocytosis of macrophages/microglia [117]. Several high-affinity specific antibodies are commercially available for the IBA1 protein. IBA1 is highly specific and capable of labeling both resting and activated microglia [118]. Since IBA1 protein is distributed in the cell body and the tiny processes of microglia, it allows the observation of morphological changes and the calculation of cell numbers (Fig. 3C). One apparent disadvantage of the IBA1 marker is that it not only labels microglia, but also macrophages, including BAMs and blood-derived monocytes [115]. Another pan-microglia marker is CX3CR1, with the same expression pattern as IBA1 [119]. CX3CR1 is a G protein-coupled receptor involved in cell adhesion and migration. However, antibodies for this protein are scarce. Instead, the reporter mouse line *Cx3cr1-EGFP* has been developed and is widely used for microglia labeling [120].

### P2RY12 and CD68

These markers are frequently used to distinguish between resting and reactivated microglia. P2RY12 is a G_i_-coupled metabotropic purinergic receptor which mediates microglial responses to extracellular nucleotides [121]. Loss of *P2ry12* in microglia switches their morphology from a highly ramified resting state to an amoeboid reactive state. Moreover, P2RY12 is strongly expressed in resting microglia, but drastically reduced after microglial activation (Fig. 3D) [121]. CD68 is a 110-kDa transmembrane glycoprotein present in monocytes and tissue macrophages [122]. It has been reported that CD68 participates in the pathological activation of macrophages by low density lipoproteins, and functions as an inhibitor of immune reactions [123]. The expression of CD68 is not conspicuous in resting microglia, but is strongly upregulated when they are activated (Fig. 3E). As a marker for reactive microglia, one disadvantage of CD68 protein is its “dotted” distribution in the membrane.

### CD206 and Hexb

Thanks to the advent of single-cell RNA sequencing technology, scientists are now able to distinguish microglia from BAMs and monocytes at the molecular level. While CD206 detects BAMs and monocytes, *Hexb* specifically labels microglia. This is an important milestone for the diagnosis of BBB damage under pathological conditions. CD206, encoded by the *Mrc1* gene, is a well-defined marker for BAMs and other macrophages without cross-labeling microglial cells (Fig. 3B) [124]. In contrast, the *Hexb* gene is a stable marker for microglia in both health and pathological conditions without discernable expression in BAMs (Fig. 3F) [125]. Thus, *Hexb* is an important marker for distinguishing microglia from surrounding macrophages. In addition, other molecular markers for CNS microglia and macrophages have also been suggested in some recent studies. For instance, TMEM119 and Siglec-H appear to be specific to microglia [126, 127], and LYVE1 preferentially labels BAMs and macrophages [128]. Unfortunately, there are no high-quality commercial antibodies for many of the aforementioned microglial markers.

## Conclusions

In general, an ideal cellular marker should be specific enough to label only one type of cell or a particular stage of cell development with high affinity. For cells of oligodendrocyte lineage, abundant stage-specific markers have been identified and high-quality antibodies have been developed accordingly. However, for astrocytes, the situation is quite different. To date, there is a lack of definitive staging for astrocyte development, partly due to the lack of stage-specific markers. Many widely-used astrocyte markers are not specific or effective enough to distinguish among RGCs, APCs, and mature astrocytes. It is now becoming clear that astrocytes, radial glial cells, and even cerebellar Bergmann cells share a similar gene expression profile, including *Glast*, *Fabp7*, *Aldh1l1*, *GFAP*, *Hopx*, *Tnc*, *Fgfr3*, *Acsbg1*, *Dbi*, and *Qk*, as well as the transcriptional factors *Sox2*, *Sox9*, *Nfia*, *Zeb1*, and *Zbtb20* [20, 40]. Although a few markers *(e.g.* S100B, GS, and NFIA) are capable of detecting most cells of astrocyte lineage, they also label other neuronal cell types such as oligodendrocytes or neurons. Therefore, a combinatory expression of multiple markers should be considered for accurate identification of astrocyte subpopulations or developmental stages.

Intriguingly, mature astrocytes share some of the same molecular marker genes (*e.g.* S100B and GS) with mature oligodendrocytes in the CNS [95, 107]. When these markers are chosen to label astrocytes, the oligodendrocyte markers SOX10, CC1, and MYRF can be used in combination for more definitive identification. This suggests that these two types of macroglia may share some common metabolic pathways. Thus, part of supporting functions of astrocytes could be taken over by myelin structures. Conceivably, myelinated axons, especially those in the white matter, are remote from their cell bodies and some metabolic support may be provided by myelin.

Another important point is that some of the markers (e.g. *Sox2*, *Fabp7*, *Sox9*, *Nfia*, *Qk*, *Zeb1*, and *Zbtb20*) expressed in RGCs and astrocytes are also maintained in OPCs, in keeping with the suggestion that RGCs are the common precursors of OPCs and astrocytes. As a matter of fact, several recent studies have identified a special class of intermediate progenitor cells (IPCs) in cortical regions that express EGFR, ASCL1, and OLIG2 during gliogenesis in both mice and primates [40, 44]. These EGFR+/ASCL1+/OLIG2+ multipotent progenitors are described as “pre-OPCs”, “pri-OPCs”, “mGPCs (multipotent glial progenitor cells)”, and “MIPCs (multipotent IPCs)” in different studies [42, 43, 129, 130]. Unexpectedly, these MIPCs are tripotent precursors as they produce not only OPCs, but also astrocytes and olfactory bulb interneurons [40]. Since the expression of OLIG2 does not restrict the fate of cells to OPCs, it seems to be more appropriate to name this type of cell “MPICs”. In light of this new finding, SOX10, rather than OLIG2, would be the best choice for labeling OPCs among glial populations in brain tissue.

Based on the similar gene expression profiling among RGCs, APCs, and astrocytes, we recently hypothesized that astrocytes are the resting neural precursor cells without undergoing terminal differentiation and cell-cycle exit, providing structural and metabolic support for local neurons [22]. Functional adaptions to the local neuronal milieu may explain the heterogeneity of astrocytes in the CNS. In support of this hypothesis, astrocytes retain some degree of stemness and reactive astrocytes are capable of differentiating into neurons or OPCs *in vitro* or *in vivo* under certain pathological conditions [131, 132]. Also, astrocytes in different regions retain a radial migration property and fail to migrate into adjacent regions after injury. Thus, astrocytes in different regions cannot be replaced by each other after lesions, contrary to the functional redundancy displayed by OPCs from different embryonic origins [19, 133].

Another issue to be aware of is the species differences in the expression of molecular markers. Although the fundamental principles are roughly the same, primates and rodents do exhibit some species-diversity in their cellular and molecular organization. For instance, cortical regions in human but not mouse develop an enlarged germinal zone called the outer subventricular zone, populated by multipotent outer radial glial cells [134, 135]. It has also been reported that GFAP in human brain and spinal cord is expressed by almost all of the astrocyte populations, while it only labels a subset of astrocytes in the murine CNS [40, 44]. Again, the classical OPC marker PDGFRA is weakly expressed in HOPX+/OLIG2+ MIPCs in the developing human cortex, but not mouse cortex before they differentiate into OPCs [44]. Therefore, it is not recommended to define a cell type with only one single marker, especially during early embryonic stages.

Methodologically, there are usually two major experimental procedures for *in situ* detection of a particular cell type in a tissue. One is through RNA *in situ* hybridization (ISH), which detects target mRNAs, and the other is by immunostaining based on specific antibody-antigen biochemical interactions. There are several advantages for ISH detection of molecular markers. First, the RNA probes for ISH are easier to prepare under laboratory conditions, especially when antibodies are not available. Second, mRNAs are usually localized to the cell body, so it is convenient for counting the number of cells of interest. Third, the difference in the nucleic acid sequence of homologous genes is greater than that of the amino-acid sequence, so the specificity of ISH is in general very high. However, its drawbacks are also apparent, as ISH is somewhat time-consuming and unsuitable for multiple-channel labeling and morphological observations. Immunostaining, including immunofluorescence, immunohistochemistry, and immunocytochemistry, is the most commonly used cell labeling technique. Immunostaining relies on specific antibodies. There are several means to validate the specificity of a particular antibody: (1) the expression pattern of a target protein detected by immunostaining should be consistent with that of its mRNA labeled by ISH; (2) knockout verification has now become a standard approach, especially when it is performed in tissue sections; (3) examination of cross-recognition between homologous proteins is recommended, considering that knockout verification of antibodies is mostly done in cell cultures, and homologous proteins that can be recognized non-specifically may not appear *in vitro*.

To date, the cellular markers for astrocytes and microglia are still relatively lacking, making it difficult to define their developmental stages, cell subtypes, or distinct functional states. At present, with the aid of single-cell RNA sequencing, it is more operable to identify novel cell type-specific markers. However, before being used as a marker, these candidate genes must be verified by both RNA *in situ* analysis and immunostaining.
